# A case of perforation of Meckel’s diverticulum with enterolith

**DOI:** 10.1186/s40792-020-00926-6

**Published:** 2020-07-06

**Authors:** Tomonari Shimagaki, Kozo Konishi, Koto Kawata, Keitaro Edahiro, Makoto Edagawa, Tomoyoshi Takenaka, Takahiro Ohmine, Nao Kinjo, Shohei Yamaguchi, Takashi Maeda, Shinichi Tsutsui, Hiroyuki Matsuda

**Affiliations:** Department of Surgery, Hiroshima Red Cross Hospital and Atomic-bomb Survivors Hospital, 1-9-6 Senda-machi, Naka-ku, Hiroshima, 730-8619 Japan

**Keywords:** Meckel’s diverticulum, Perforation, Enterolith

## Abstract

**Background:**

Perforation of Meckel’s diverticulum with enteroliths is a rare complication. Here, we report a case of perforation of Meckel’s diverticulum with one enterolith, which could not be accurately diagnosed by preoperative computed tomography.

**Case presentation:**

A 16-year-old male patient with acute onset of severe abdominal pain and a localized muscle guarding in the right hypochondrium had a solitary stone detected in the right abdomen by radiography. Abdominal computed tomography revealed a saclike outpouching of the small intestine, which contained fluid levels and an enterolith, with a mesenteric inflammatory change in the right paraumbilical area. He was diagnosed with peritonitis due to appendicitis or Meckel’s diverticulitis with enterolith, and emergency operation was indicated. The perforated Meckel’s diverticulum was identified approximately 30 cm proximal to the ileocecal valve. The diverticulum was transected at the base and removed. The patient’s postoperative course was uneventful.

**Conclusions:**

It is crucial for clinicians to thoroughly examine patients and appropriately request investigations that consider perforation of Meckel’s diverticulum as a possible diagnosis to facilitate prompt treatment.

## Background

The German anatomist Johann Friedrich Meckel first described the embryological and pathological features of Meckel’s diverticulum in 1809 [1]. Meckel’s diverticulum is the most prevalent congenital abnormality of the gastrointestinal tract, occurring in approximately 2% of the general population. When present, there is an estimated 4–16% lifetime risk of becoming symptomatic, including bleeding, intussusception, inflammation, and occasionally perforation [2, 3]. However, the formation of enteroliths in the diverticulum is an uncommon condition. Meckel’s diverticulitis is difficult to diagnose preoperatively and is often diagnosed only after laparotomy. Here, we report a rare case of perforation of Meckel’s diverticulum with an enteric calculus.

## Case presentation

A 16-year-old male patient with no prior abdominal surgery presented to our department with 2 days of abdominal pain. He complained of nausea and vomiting. He had had occasional lower abdominal pain for 7 years prior to presenting to our department. Physical examination of the patient revealed abdominal tenderness and localized guarding in the right hypochondrium. The vital signs were a bit unstable, with a blood pressure of 98/60 mmHg, heart rate of 102 beats/min, and body temperature of 39.6 °C. The blood test revealed a raised white blood cell count of 18,900/μl and a high C-reactive protein level of 4.77 mg/dl. The patient had normal renal function and a normal hemoglobin level. The laboratory findings upon admission are shown in Table 1.

**Table 1:**
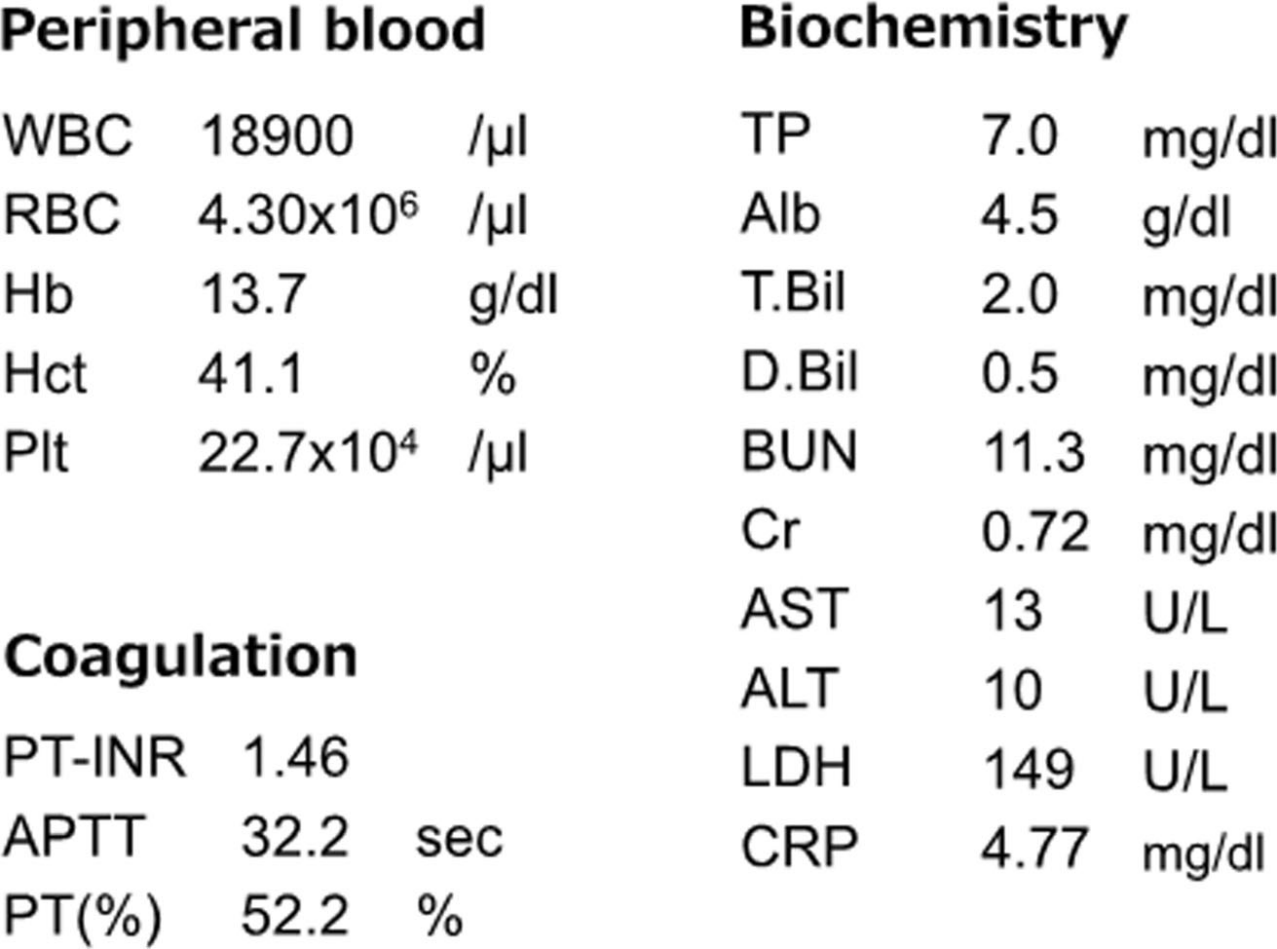
Laboratory findings upon admission

**Fig. 1:**
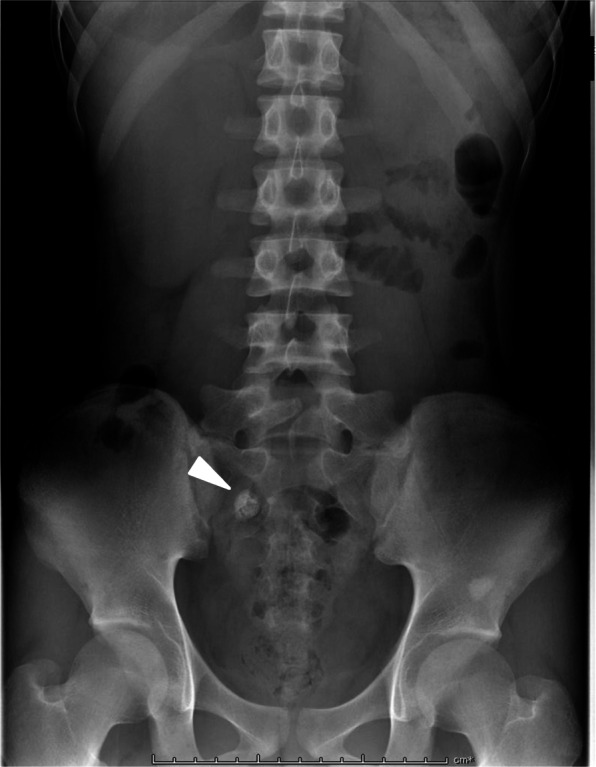
Preoperative abdominal radiography. A solitary stone with peripheral calcification and a lucent center was identified on abdominal radiography (arrowhead)

Abdominal radiography showed a solitary stone with peripheral calcification and a lucent center in the right abdomen (Fig. 1).

**Fig. 2:**
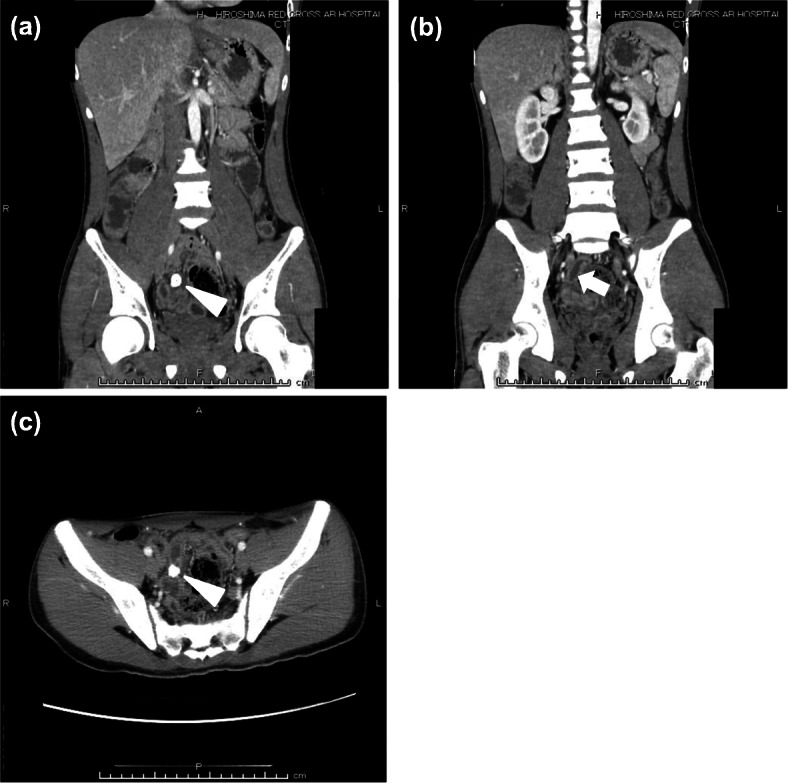
Preoperative enhanced computed tomography (CT) images. Enhanced **a**, **b** coronal and **c** axial CT images showed an enterolith (arrowhead) in a tubular blind-ending structure, with a thickened and hypercaptant wall (arrow)

Abdominal computed tomography (CT) confirmed that niveau formation and a substance with calcification in the outer shell were observed in the extended intestine in the lower abdomen (Fig. 2a–c). The patient was diagnosed with peritonitis due to appendicitis or Meckel’s diverticulitis with enterolith, with ascites in the rectovesical pouch, and emergency surgery was performed.

**Fig. 3:**
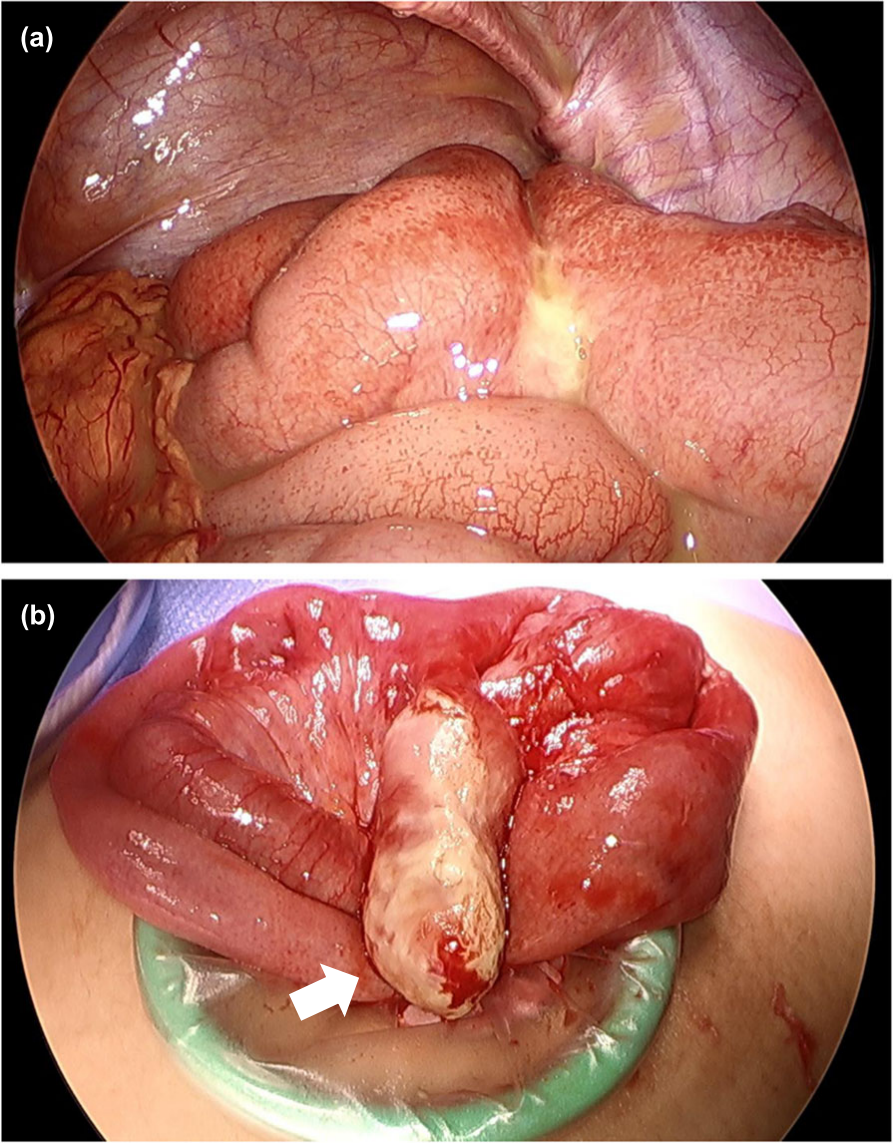
Intraoperative findings. **a** A small amount of turbid ascites was found in the abdominal cavity. **b** Surgical appearance of Meckel’s diverticulum (arrow) approximately 30 cm proximal to the ileocecal valve

When the operation was started with single-port laparoscopic surgery, a small amount of turbid ascites was found in the abdominal cavity (Fig. 3a). The appendix showed redness on the serosal surface, with mild inflammation. The appendix was resected. As we explored further, the perforated Meckel’s diverticulum containing the enterolith was identified approximately 30 cm proximal to the ileocecal valve. The base of the diverticulum was stenotic, and a perforation was observed in the vicinity (Fig. 3b). The Meckel’s diverticulum was dissected from the mesentery and transected at its base. The patient’s postoperative course was uneventful.

**Fig. 4:**
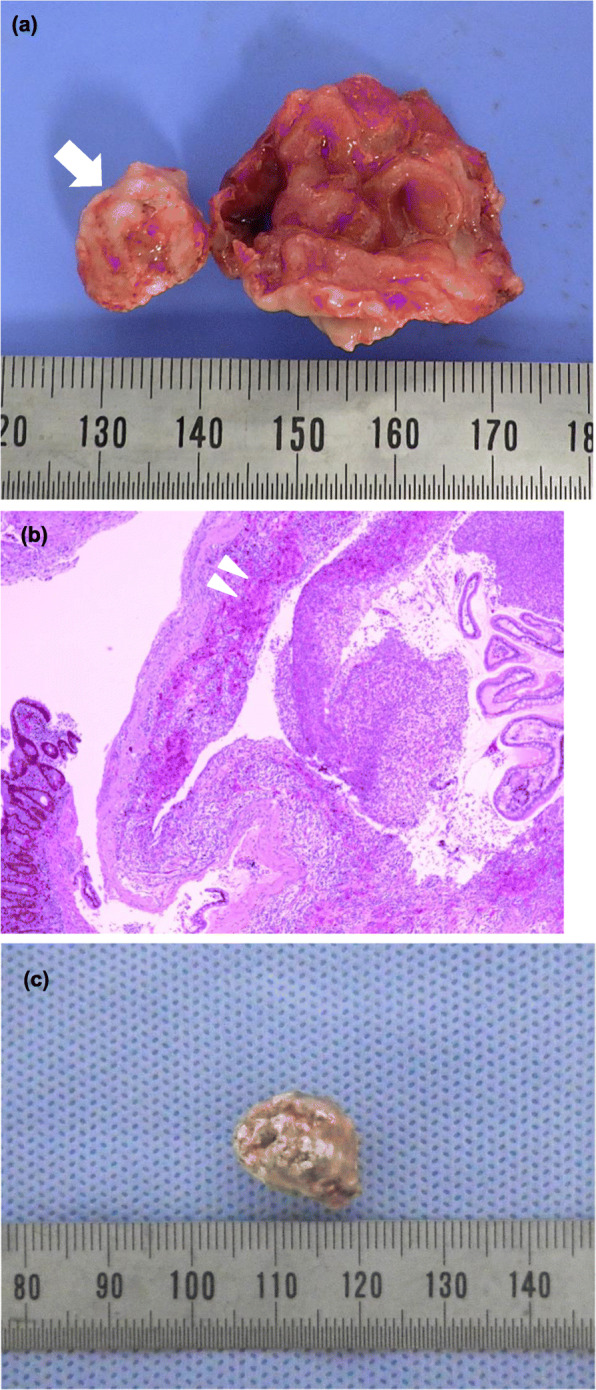
Postoperative specimen findings. **a** Macroscopic findings of the surgical specimen of the Meckel’s diverticulum showed a narrow-necked diverticulum containing one enterolith (arrow). **b** Microscopic findings of mucosal inflammation and hemorrhage (arrowhead) at the site of the perforation (H&E × 40). **c** A photograph of this enterolith

The macroscopic findings of the surgical specimen of the diverticulum confirmed a narrow-necked diverticulum lined by noninflamed small bowel mucosa containing one enterolith (Fig. 4a). Microscopic examination revealed severe mucosal inflammation and hemorrhage without surrounding ectopic gastric mucosa around the perforation (Fig. 4b). The component of the enterolith was calcium phosphate stone (Fig. 4c). In addition, the resected appendix showed reactive lymphoid hyperplasia during microscopic examination.

## Discussion

Meckel’s diverticulum is the most common congenital abnormality in the gastrointestinal tract, occurring in 2% of the population, with a 4.2 –6.4% risk of complication [4]. The omphalomesenteric duct is the point of original communication between the yolk sac and the bowel lumen, which typically closes during the 9th week of gestation. A Meckel’s diverticulum is formed when the proximal portion of the omphalomesenteric duct fails to completely obliterate.

Enteroliths within Meckel’s diverticulum are thought to be rare; however, the incidence is difficult to ascertain because its existence has only been documented in case reports. Primary enteroliths are thought to result from precipitation and deposition of enteric contents associated with hypomotility and stasis, whereas secondary enteroliths include foreign bodies that clump together and serve as a nidus for intestinal content deposition [5]. It is thought that the alkaline environment of the small bowel favors precipitation [5]. Enteroliths within or expelled from a Meckel’s diverticulum can be asymptomatic [6] or present with perforation [7], diverticulitis, or bowel obstruction [8]. Including Fig. 3 in this study, only a few operative photos of enteroliths arising from Meckel’s diverticulum have been published [8].

Meckel’s diverticulum perforation is a serious and often life-threatening complication, usually secondary to diverticulitis, gangrene, or peptic ulceration due to ectopic gastric mucosa. Other various pathologies leading to perforation are Littre’s hernia and tumors such as leiomyosarcoma, lymphatic sarcoma, and poorly differentiated stromal tumors [9]. Perforation of Meckel’s diverticulum by foreign bodies is extremely rare, and in a review, the indication rate for a resection due to perforation by a foreign body like fish bone and toothpick was reported to be 8% of all complicated diverticula [10]. Chan et al. reported more than 300 cases of perforation of Meckel’s diverticulum by foreign body [11], and among them, cases of fish and chicken bone, wood splinter, and button battery have been mentioned in the literature [11, 12]. It is said that the tendency of these objects to lodge in the blind pouch of the Meckel’s diverticulum causes the perforation. In our present case, only a few cases of perforation at the site of the diverticulum with true enterolith formation like the calcium phosphate stone have been reported in the literature [13, 14]. One possible explanation of this rarity is that most Meckel’s diverticula have wide necks and contain smooth muscle capable of peristalsis [2]. Therefore, stagnation and stasis of the intestinal contents is unlikely. The pathogenesis of true enterolith formation in Meckel’s diverticulum is unclear. It may be related to stasis resulting from poor coordination of the peristaltic wave at the site of the Meckel’s diverticulum [6]. Also, superimposed inflammation and edema of the neck of the diverticulum could narrow the opening and decrease drainage, leading to precipitation and nidus formation of the true enterolith. Approximately one-third of enteroliths were radiopaque and therefore demonstrable radiographically like in our present case [13].

A symptomatic or complicated Meckel’s diverticulum diagnosis is difficult to confirm on the basis of history, physical examination, laboratory findings, and imaging because a variety of conditions mimic Meckel’s diverticulum both clinically and radiologically (such as appendicitis, ileal/colonic diverticulitis, or regional enteritis/colitis) [4]. Traditionally, clinicians confronted with a patient with complicated Meckel’s diverticulum relied on conventional gastrointestinal contrast studies, angiography, or scintigraphy. However, these methods have been progressively replaced by CT, which is now routinely used as the first-line imaging tool in the diagnostic workup of the acute abdomen [15]. The sensitivity of diagnosing Meckel’s diverticulum on CT scan has increased owing to the availability of higher spatial resolution and the multiplanar isotropic reconstruction ability of the latest Meckel’s diverticulum CT scanners, which allow visualization of the small bowel in various planes [4].

Management of symptomatic or complicated Meckel’s diverticulum is generally through open or laparoscopic resection of the diverticulum. With respect to this specific complication, several approaches have been described, including milking the enterolith distally into the cecum or proximally into a healthy small bowel and performing an enterotomy to extract it [16]. Alternatively, resection of the segment of the small bowel containing the Meckel’s diverticulum and enterolith followed by primary anastomosis can be performed [3]. In this case, a single-port laparoscopic procedure successfully confirmed and resected the Meckel’s diverticulum with enterolith.

## Conclusions

If an inflammatory process is visualized on CT in the lower abdomen or pelvis, particularly at midline, or if there is evidence of distal small bowel obstruction, one should carefully search for the presence of a complicated diverticulum. If a normal appendix is identified, the likelihood of this diagnosis increases. Meckel’s diverticulum complications present with a wide range of clinical and imaging manifestations, from benign indolent findings to acute life-threatening conditions. CT findings of complicated Meckel’s diverticulum are very polymorphic and should be considered during the evaluation.

## Data Availability

Not applicable.

## Abbreviation

CT: Computed tomography
